# The Origin of Dielectric Permittivity in Plants

**DOI:** 10.3390/ijms27135735

**Published:** 2026-06-25

**Authors:** Festa Margherita, Pianta Marta, Miskovsky Pavel, Niaz Esha, Anguera Jaume, Roccotiello Enrica, Carpaneto Armando

**Affiliations:** 1 Department of Earth, Environment and Life Sciences (DISTAV), University of Genoa, Viale Benedetto XV 5, 16132 Genova, Italy; margherita.festa@edu.unige.it (F.M.); marta.pianta@edu.unige.it (P.M.); esha.niaz@edu.unige.it (N.E.); 2 Technology Department, Ignion, 08174 Barcelona, Spain; pavel.miskovsky@ignion.io (M.P.); jaume.anguera@ignion.io (A.J.); 3 Telecommunication Engineering, Universitat Ramon Llull, 08022 Barcelona, Spain

**Keywords:** permittivity, dielectric spectroscopy, dispersion, loss tangent, polarization, plant physiology

## Abstract

Dielectric permittivity describes how a material becomes polarized in response to a time-varying electric field and provides a powerful framework for probing the physical organization of biological systems. This review aims to clarify the origin of dielectric permittivity in plants, offering a conceptually grounded interpretation while keeping mathematical formalism to the level necessary for biological interpretation. We first outline the fundamental mechanisms of polarization, their characteristic time scales, and the frequency-dependent nature of the dielectric response, including the concept of complex permittivity, together with commonly used measurement approaches in biological materials. Particular attention is given to water, whose dielectric properties play a dominant role in plant tissues. We then examine how permittivity varies across different plant organs, including leaves, fruits, and roots, highlighting the relationship between dielectric response and structural and compositional features. Modeling strategies linking microscopic organization to macroscopic dielectric behavior are also discussed. Because dielectric permittivity is intrinsically connected to plant structure and composition, non-invasive measurements offer significant potential for assessing plant physiological status, including the detection of changes induced by abiotic and biotic stresses. By bridging engineering approaches with plant physiology, this review provides a unified framework to interpret dielectric measurements in plants and supports their application in plant science and phenotyping.

## 1. Introduction

Living organisms exploit electrical phenomena in multiple functional contexts relevant to their survival. Across the biological membrane enclosing cells, charge separation gives rise to a membrane potential which, in plants, can reach values down to approximately −200 mV, with negative charges localized on the cytosolic side; the movement of charges across plant cell membranes is mediated by the activity of specific ion transporters and ion channels selective for cations and anions [1]. More recently, electron transport mediated by specific cytochromes has also been directly measured [2,3].

From a biophysical perspective, electrical measurements provide access to key structural and functional membrane properties. One of the earliest determinations of membrane thickness was derived from capacitance measurements by Fricke [4]. Using a non-invasive analysis, Fricke obtained an estimate of the membrane-specific capacitance of erythrocytes on the order of 1 µF/cm^2^. This was achieved by analyzing the frequency-dependent dielectric response of diluted cell suspensions, modeled as insulating shells enclosing conductive interiors, and interpreting the data within an approach that treats the heterogeneous system as an equivalent homogeneous material, enabling the separation of bulk conduction and interfacial polarization contributions, thereby allowing for the calculation of the membrane capacitance. Comparable values of specific membrane capacitance have since been confirmed across a wide range of cell types [5]. An estimate of membrane thickness follows directly from the parallel-plate capacitor model, C = ε_0_ε_r_A/d, where C is the total membrane capacitance, A the membrane area, d the membrane thickness, ε_0_ the vacuum permittivity, and ε_r_ the relative permittivity of the phospholipid bilayer. Since the specific membrane capacitance C_s_ = C/A = 1 µF/cm^2^, and assuming a phospholipid relative permittivity ε_r_ between 2 and 3, the membrane thickness is obtained as d = ε_0_ε_r_/C_s_, yielding values in the nanometer range, consistent with the typical thickness of lipid bilayers.

These classical electrochemical and dielectric approaches naturally extend to plant systems, where permittivity emerges as a multiscale property rather than a purely cellular parameter. In plants, dielectric permittivity arises from the hierarchical organization of tissues, cells, membranes, intercellular spaces, and interfaces, and therefore provides integrated information on structure and physiological state across multiple spatial scales. A central element linking all these contributions is water, which acts as the dominant polar component in living plant tissues and largely governs their dielectric response.

The aim of this review is to discuss the physical origin of dielectric permittivity in plant systems, its frequency-dependent behavior, and its relationship with plant organization, physiology, and emerging bioelectromagnetic applications.

## 2. Material Permittivity: Definition, Mechanisms and Measurement

The starting point to understand permittivity is the parallel-plate capacitor. It consists of two conducting plates of area A separated by a distance d. When equal and opposite charges ±Q are placed on the plates spaced by a vacuum, an electric field E is established in the region between them. From Gauss’s law, the field is uniform and depends on the surface charge density, giving E = σ/ε_0_, where σ = Q/A. By integrating the electric field across the separation distance, the potential difference is obtained as ΔV = ∫E·dl = (σ/ε_0_)d = (Qd)/(ε_0_A). This leads directly to a proportional relationship between charge and voltage, written as Q = C·V, with the capacitance of the parallel-plate capacitor given by C = ε_0_A/d. The concept of permittivity naturally extends from membrane capacitance: when a non-conducting material (a dielectric) is inserted between the plates, the system changes. Under the action of the electric field, the dielectric becomes polarized. As a result, for the same amount of charge on the plates, the internal electric field is reduced because polarization charges appear within the material and partially oppose the applied field. This leads to an increase in capacitance by factor ε_r_, the relative permittivity, so that C = ε_0_·ε_r_·A/d. In air, ε_r_ = 1; thus, ε_r_ quantifies how strongly a material enhances the capacitance relative to vacuum.

### 2.1. Mechanism of Polarization

In apolar dielectrics, polarization can be understood as a slight displacement of the electron cloud of each atom with respect to its positively charged nucleus; this type of polarization is known as electronic polarization. In biological materials, the corresponding relative permittivity values are typically low, usually in the range εr ≈ 2–4; for example, the hydrophobic core of biological membranes, composed of lipid acyl chains, represents a prototypical apolar dielectric with a relative permittivity in this range [6].

The second type of polarization is atomic (or ionic) polarization [7]. Here, ions (nuclei together with their bound electrons) are displaced relative to each other under the applied field. This can be viewed as an elastic displacement of charged entities within the material. Typical contributions lie in the range of ε_r_ ≈ 2–10, depending on material structure.

The third type of polarization is dipolar orientational polarization, which occurs in materials with permanent dipoles. A classic example is water, where molecular dipoles tend to rotate and align with the applied field. This leads to a stronger dielectric response, with ε_r_ ≈ 80.

The fourth type of polarization is interfacial polarization, also known as Maxwell–Wagner polarization [8]. It occurs in heterogeneous systems due to charge accumulation at interfaces between regions with different electrical properties (e.g., conductivity and/or permittivity). In biological cells, charge separation occurs mainly across the cell membrane, which acts as a thin insulating layer between conductive intracellular and extracellular media. Charges accumulate on both sides of the membrane, producing a strong interfacial polarization. Consequently, the effective permittivity can reach values of ε_r_ ≈ 10^3^–10^4^. In tissues, this effect is exacerbated by the presence of many cells and interfaces. The accumulation of charge at multiple boundaries leads to even higher effective permittivity values, typically ε_r_ ≈ 10^5^–10^6^. It is worth noting, however, that strictly speaking, these permittivity values do not depend on the dielectric nature of the material, but rather on the mobility of internal mobile charges.

### 2.2. Relaxation Time Scales

The characteristic time of a polarization mechanism, τ, is defined as the time required for the polarization to adjust to a change in the applied electric field [9]. When the field is switched on or varies, the system does not respond instantaneously but relaxes toward equilibrium over a finite time.

For electronic polarization, the moving charges are electrons. The relevant scale is atomic, with very small mass and strong Coulomb forces, leading to $\tau \approx 10^{-16}\;s$.

For atomic polarization, the motion involves ions (nuclei with bound electrons). The system behaves like an elastic lattice with much larger masses and restoring forces, giving $\tau \approx 10^{-14}-10^{-13}\;s$.

For dipolar orientational polarization, the moving entities are polar molecules such as water. The process involves rotational motion in a viscous environment, influenced by thermal collisions, with $\tau \approx 10^{-11}-10^{-9}\;s$.

For interfacial polarization, charge accumulation occurs at boundaries. The mechanism is governed by charge transport processes such as conduction and diffusion in heterogeneous media, typically with $\tau \approx 10^{-3}-10^{-1}\;s$.

The permittivity of a biological organ or tissue results from the combined contribution of all these polarization mechanisms.

When an alternating electric field is applied, the characteristic relaxation times τ of the dielectric polarization mechanisms described above lead to frequency-dependent responses. Each mechanism is associated with a characteristic relaxation frequency $f_{c}=\frac{1}{2\pi \tau }$, as described by Debye-type relaxation models (see Section 5). For frequencies $f\ll f_{c}$, polarization can follow the applied field and contribute fully to the permittivity. For $f\gg f_{c}$, the mechanism cannot respond effectively, and its contribution to the dielectric constant decreases. At $f=f_{c}$ the system undergoes a transition between the low-frequency and high-frequency regimes, giving rise to so-called dielectric dispersion centered around the characteristic relaxation frequency. Overall, the frequency dependence of these polarization mechanisms underlies dielectric dispersion in materials [10,11].

### 2.3. Complex Permittivity and Frequency Response

When a dielectric is placed between the plates of a capacitor, its capacitance changes; these variations can be used experimentally to determine the permittivity. Under a sinusoidal applied field, the response is characterized by amplitude and phase. For this reason, a complex representation is required. Without entering mathematical details, the dielectric permittivity at a given frequency is described by two measurable quantities: ε′ and ε″. ε′ is the real part (reactive component), representing energy stored in the material. It quantifies how effectively polarization follows the applied field and corresponds to reversible (elastic) energy storage. In the static limit, ε_r_ = ε′ because no losses are present (ε″ = 0). Thus, ε′ describes how strongly the material polarizes in phase with the field; ε″ is the imaginary part (dissipative component), associated with energy losses. It accounts for frictional motion of dipoles, finite conductivity, and delays in charge transport, all of which convert electrical energy into heat. It therefore quantifies the energetic cost for the material to follow the field.

A useful physical picture is to compare an ideal capacitor, where energy is stored without loss, with a resistor, where charge motion leads to Joule heating and energy dissipation.

The frequency-dependent behavior of ε′ and ε″ defines the dielectric spectrum of the material. Often, instead of ε″, the loss tangent tanδ = ε″/ε′ is reported as a function of frequency.

In summary, ε′ and ε″ together provide a complete description of the electromagnetic response of a material.

### 2.4. Permittivity Measurement Methods in Plant Systems

In the frequency range from quasi-static conditions up to ~10 GHz, permittivity measurements in biological systems rely on techniques that combine broadband capability with compatibility with hydrated and heterogeneous samples. At low frequencies (Hz–MHz), lumped-element methods such as parallel-plate capacitors and impedance spectroscopy are commonly used, treating the sample as an equivalent circuit element [12].

At higher frequencies (MHz–GHz), transmission and reflection techniques are preferred, including coaxial probes and transmission-line or waveguide configurations, where permittivity is extracted from scattering parameters (S-parameters). Open-ended coaxial probes are particularly suitable for biological tissues because they require minimal sample preparation and can be applied directly to hydrated materials [13]. However, their accuracy depends critically on the quality of contact between the probe aperture and the sample. A small air gap between the probe and the tissue introduces a low-permittivity layer at the measurement interface and can therefore bias the extracted permittivity, usually toward lower apparent values. This issue is particularly relevant for heterogeneous plant surfaces (see Table 1), such as leaves, stems, bark, fruits, and roots, where curvature, roughness, epidermal structures, trichomes, or waxy layers may prevent complete contact. For this reason, the probe is commonly pressed gently against the sample to eliminate air gaps and improve repeatability. Nevertheless, excessive pressure can deform soft biological tissues, alter the local water distribution, squeeze intercellular or extracellular fluid, and change the volume effectively sensed by the evanescent field. Contact pressure should therefore be minimized, standardized, and reported when possible, for example, by using a mechanical holder or spring-loaded fixture. This limitation is especially important for plant tissues with high turgor pressure, large intercellular spaces, or easily deformable parenchyma, because compression may modify the dielectric response being measured. Resonant methods provide high accuracy at discrete frequencies but are less suited to heterogeneous samples, while time-domain techniques offer broadband characterization [8]. Overall, method selection is constrained by the need to preserve physiological conditions, avoid sample deformation, and account for high water content, conductivity, and tissue heterogeneity [13,14,15].

Beyond methodological aspects, the measured dielectric permittivity is affected by a combination of physical parameters and biological factors, including temperature, hydration status, and structural features, as summarized in Table 2.

## 3. Permittivity of Water

Because water represents one of the dominant contributors to plant dielectric behavior, understanding its relaxation dynamics is essential for interpreting permittivity measurements in plant tissues and their physiological responses.

**Figure 1:**
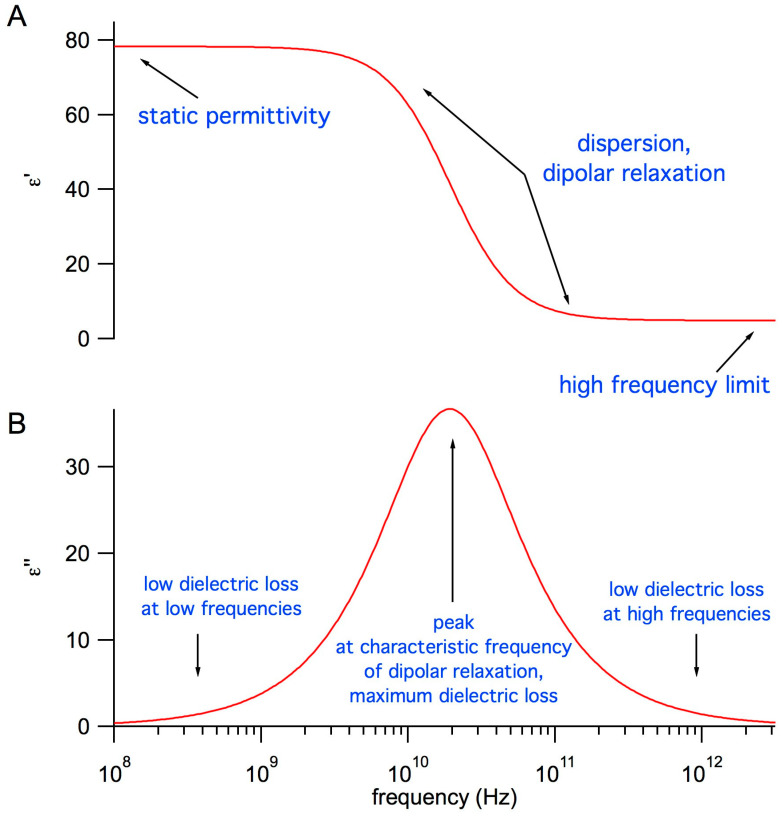
Real and imaginary parts of the complex permittivity of pure water at 20 °C calculated using the Debye relaxation model (see Section 6, Equation (1)). The parameters used are ε_s_ = 78.3, ε_∞_ = 4.9 and τ = 8.27 10^−12^ s. (**A**) Frequency dependence of the real part of the permittivity (ε′, see Section 6, Equation (2)), showing a monotonic decrease from the static permittivity (ε_s_) at low frequencies toward the high-frequency limit (ε_∞_) as frequency increases. (**B**) Frequency dependence of the imaginary part of the permittivity (ε″, see Section 6, Equation (3)), exhibiting a pronounced relaxation peak centered at the characteristic frequency f_c_ = 1/2πτ = 19.3 GHz, corresponding to maximum dielectric loss associated with dipolar relaxation processes.

The permittivity of water arising from the reorientation of molecular dipoles is illustrated in Figure 1. Water molecules are strong dipoles and, under an applied electric field, they tend to rotate; thus, dipolar rotation is the dominant mechanism of polarization. In Figure 1A, the real part of the permittivity (ε′) is shown as a function of frequency. At low frequencies, the dipoles can follow the applied field, resulting in a high value of ε′ of about 80. At frequencies above ~1 GHz, ε′ decreases, indicating that the dipoles can no longer follow the field efficiently. As stated in the previous section, this decrease is referred to as dispersion (see below for the γ-dispersion). At frequencies above ~100 GHz, ε′ reaches a minimum, reflecting the fact that dipolar motion is no longer able to respond to the rapidly varying field. Figure 1B shows the imaginary part of the permittivity (ε″). At frequencies below ~1 GHz, dipoles follow the field effectively, and dissipation is minimal, resulting in low ε″ values. At a characteristic frequency of approximately 17 GHz (f = 1/2πτ, with τ ≈ 8 ps), ε″ exhibits a peak: dipoles attempt to follow the field but lag behind it, leading to maximal energy dissipation (i.e., maximum “dynamic friction”). At higher frequencies (>100 GHz), dipoles are no longer able to reorient, and ε″ decreases again. The peak in ε″ therefore corresponds to the frequency at which the system experiences the greatest difficulty in following the applied field.

This behavior can be generalized: when ε′ decreases (dispersion), ε″ exhibits a maximum. In this sense, ε′ reflects how effectively polarization follows the applied field, whereas ε″ quantifies the energy dissipated when the polarization cannot keep up with field variations.

### 3.1. Free Water, Bound Water and Hydrated Water

In plant tissues, water does not behave as a single homogeneous dielectric phase. Instead, it exists in different physical states that contribute differently to the measured complex permittivity. A useful distinction can be made among free water, bound water, and hydration water associated with ions and charged macromolecular surfaces.

Free water is the fraction of water that is relatively mobile within vacuoles, cytoplasm, apoplast, xylem vessels, intercellular spaces, and extracellular aqueous compartments. Its dielectric behavior is closest to that of bulk liquid water, described above, with a high real permittivity at low microwave frequencies and a dominant dipolar relaxation in the GHz range. Because of its high dielectric strength, changes in free-water content strongly affect ε′, especially in radiofrequency and microwave measurements. Thus, tissue dehydration, drought stress, reduced irrigation, or loss of turgor generally decrease the contribution of free water and therefore reduce the apparent permittivity. Conversely, rehydration or tissues with high succulence usually show larger dielectric loading.

Bound water refers to water molecules whose rotational mobility is restricted by hydrogen bonding, adsorption, confinement, or interaction with macromolecular surfaces. In plants, bound water is associated with cell-wall polysaccharides such as cellulose, hemicellulose and pectins, proteins, membranes, starch granules, and other hydrophilic or charged structures. Compared with free water, bound water has a lower effective dielectric strength and a broader, slower relaxation because its dipoles cannot rotate as freely. In leaf tissues, bound water has been associated with water physically or chemically bound to cell walls and organic compounds, and its characteristic relaxation has been reported at a much lower frequency than that of free water [16]. Therefore, an increase in the relative fraction of bound water, as can occur during dehydration or osmotic adjustment, does not simply reproduce the dielectric behavior of bulk water. Instead, it broadens the dielectric spectrum and shifts part of the water-related response toward lower frequencies. This is one reason why plant tissues rarely follow a single-Debye relaxation model (see Section 5).

Hydrated ions provide an additional contribution. Dissolved ions such as K^+^, Ca^2+^, Mg^2+^, Cl^−^, nitrate, phosphate, and organic counterions are surrounded by hydration shells in which water mobility is reduced by ion–water interactions. Dielectric relaxation studies of aqueous electrolyte solutions show that ions modify both the relaxation behavior and the static permittivity of water, with solvation effects contributing to the concentration dependence of the dielectric response [17]. When salts are added to pure water, only moderate changes in ε′ and ε″ are observed. In particular, the low-frequency plateau of ε′ decreases, and the peak of ε″ is reduced. For example, at 1.42 M KCl, the maximum value of ε′ decreases from ~78 to ~65, while the peak of ε″ decreases from ~35 to ~30 [18]. At the same time, ions increase electrical conductivity, introducing an additional contribution to ε″ at low and intermediate frequencies. Therefore, salinity, nutrient status, membrane leakage, and stress-induced changes in ion transport can modify dielectric spectra through two coupled mechanisms: changes in dipolar water relaxation and changes in conductive/interfacial losses [16,17]. In living tissues, these effects are difficult to separate because water content, ion concentration, membrane integrity, and tissue structure change simultaneously.

From a physiological perspective, drought and water-stress responses alter all three water fractions. Loss of free water reduces dielectric strength; osmotic adjustment and accumulation of soluble compounds increase the fraction of hydration water; membrane damage or altered ion-channel activity changes ionic conductivity; and changes in root water uptake or hydraulic redistribution modify spatial water distribution. Consequently, dielectric measurements can provide a non-invasive signature of plant water status, but the measured permittivity should be interpreted as an integrated response of water amount, water mobility, ion content, and tissue compartmentalization rather than as a direct measurement of total water content alone [19,20,21].

### 3.2. Novel Antenna Designs

Interestingly, the frequency-dependent permittivity of water has enabled novel antenna designs in the microwave domain. At microwave frequencies, water exhibits a relatively high dielectric permittivity, which makes it suitable as a radiating or loading material for compact antenna structures. Simultaneously, water is optically transparent in the visible spectrum, allowing for the transmission of light through the antenna volume.

This unique combination of electromagnetic and optical properties enables the integration of photovoltaic cells beneath water-based antennas, leading to compact multifunctional systems. In contrast, conventional metallic antennas fabricated from conductive materials such as copper are opaque to visible light, preventing the placement of solar cells directly underneath the radiating element without significantly blocking incident illumination. Therefore, water-based antennas provide a promising approach for co-locating wireless communication and solar-energy-harvesting functionalities within the same physical footprint [22,23].

## 4. Plant Dielectric Permittivity Across Scales

Plant tissues represent highly heterogeneous biological systems in which water content, membrane organization, ionic composition, and structural compartmentalization coexist across multiple spatial scales. Consequently, their dielectric properties emerge from the combined contribution of different polarization mechanisms associated with tissues, cells, membranes, and intracellular/extracellular fluids [24,25]. Measuring permittivity in plant systems is therefore of considerable interest because it provides a non-destructive and potentially rapid approach to investigate tissue organization, hydration status, membrane integrity, and physiological responses to environmental stress [26,27,28]. Variations in dielectric behavior have been associated with changes in water availability, salinity, temperature stress, and tissue viability, making dielectric spectroscopy a promising tool for plant phenotyping and physiological monitoring [29].

Recent studies have also highlighted the relevance of plant dielectric properties in emerging bioelectromagnetic and wireless communication applications, where leaf tissues can directly interact with radiofrequency signals and antenna systems [30,31]. In these systems, the electromagnetic response of leaves appears to depend on several structural and functional traits, including hydration status, nitrogen content, tissue conductivity, leaf thickness, and internal anatomical organization, all of which may contribute to variations in dielectric permittivity, dielectric losses, impedance, and electromagnetic coupling [16,31,32,33].

### 4.1. Structural Organization and Functional Traits

Comparative analyses among species further suggest that dielectric behavior is strongly influenced by plant functional and anatomical traits. Parameters such as leaf thickness, water content, tissue compactness, cuticle properties, venation density, stomatal distribution, surface roughness, trichome presence, mesophyll organization, conductivity, ionic composition, and the proportion of intercellular air spaces may all contribute to variations in dielectric response across frequencies [34,35,36]. Functional traits related to stress adaptation, including leaf dry matter content, succulence, sclerophylly, tissue hydration dynamics, and nitrogen concentration, may further modulate polarization mechanisms associated with membranes, interfaces, ionic transport, and water organization within tissues [37,38].

Among these factors, water content likely plays a dominant role because of the high dielectric permittivity and dielectric loss associated with dipolar relaxation processes. At the same time, tissue thickness and conductivity may influence dielectric loading and electromagnetic interactions in radiofrequency applications [16,31,32,33]. Overall, dielectric spectra should therefore not be interpreted only phenomenologically, but also in relation to the structural and functional traits underlying dielectric diversity among plant tissues and their responses to environmental conditions.

In addition to studies reporting complete dielectric spectra across broad frequency ranges, many investigations have focused on selected frequency windows associated with specific biological or technological applications, including hydration monitoring, stress detection, fruit ripening assessment, root activity estimation, and microwave sensing. Consequently, the dielectric parameters reported in the literature span different species, tissues, physiological conditions, and frequency domains. Together, these studies highlight the large variability in dielectric properties among plant taxa and support the hypothesis that structural and functional traits contribute substantially to the observed dielectric diversity.

### 4.2. Frequency-Dependent Dielectric Behavior in Plants

**Figure 2:**
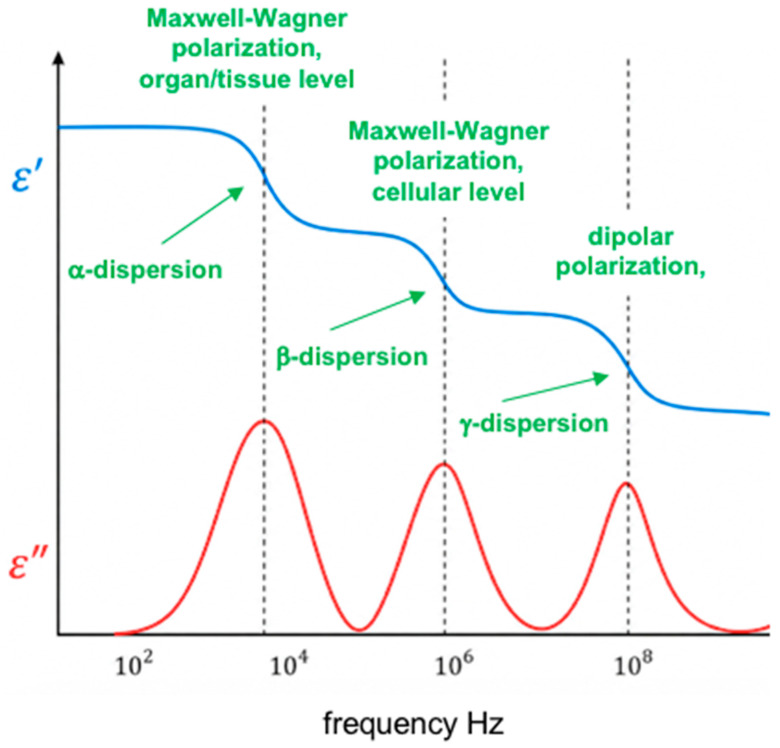
Typical frequency-dependent profile of the complex permittivity in plant systems. The real part of the permittivity (ε′, blue curve) shows a stepwise decrease with increasing frequency, reflecting successive polarization mechanisms with different characteristic relaxation times. The imaginary part (ε″, red curve) exhibits multiple relaxation peaks, each associated with distinct dielectric dispersion processes occurring in biological tissues.

From a general point of view, the spectrum of ε′ as a function of frequency (Figure 2) shows a sequence of step-like decreases. These steps correspond to the progressive deactivation of different polarization mechanisms: as the frequency increases, each polarization process is no longer able to follow the oscillations of the external electric field and therefore progressively drops out of the dielectric response.

The dashed vertical lines indicate representative frequency windows (from ~10^2^ to ~10^12^ Hz) where different polarization mechanisms dominate, including interfacial (Maxwell–Wagner), ionic, dipolar (water-related), and high-frequency molecular contributions. The overall behavior highlights the broadband and multi-relaxation nature of plant dielectric response, which deviates from single-Debye behavior due to structural heterogeneity and water content distribution within tissues.

In parallel, the ε″ spectrum exhibits a series of peaks, each one associated with a dielectric dispersion process. These peaks correspond to energy dissipation mechanisms linked to the relaxation of the different types of polarization.

Overall, the dielectric response of plant tissues can be divided into several frequency-dependent regimes, commonly referred to as the α, β, and γ dispersions, each reflecting a different physical scale of interaction between the electromagnetic field and the biological structure (see Table 3).

In the low-frequency range (Hz–kHz), corresponding to the α-dispersion, the response is dominated by large-scale interfacial polarization processes, typically described as Maxwell–Wagner polarization at the organ or tissue level. In this regime, charge carriers have sufficient time to accumulate at interfaces such as cell walls, cell–cell boundaries, and extracellular spaces, as well as along complex conductive pathways within the tissue. Therefore, a strong charge separation builds up at macroscopic heterogeneities, leading to very large effective permittivity values (ε′), which can range from 10^2^ up to 10^4^ or even higher depending on structural complexity and hydration.

When moving to intermediate frequencies (kHz–MHz range), corresponding to β-dispersion, the dielectric response becomes dominated by processes occurring at the cellular level. This regime is generally associated with Maxwell–Wagner polarization across cell membranes and is strongly related to the electrical properties of individual cells. The cell membrane plays a central role here and can be modeled as a capacitor due to the amphipathic nature of phospholipids. These molecules form a bilayer structure in which hydrophobic tails face inward, creating an insulating layer that allows for charge accumulation on both sides of the membrane. In this frequency range, ε′ decreases with respect to the α-regime but remains relatively high (typically between ~10^2^ and 10^3^ depending on tissue type and physiological state). β-dispersion is therefore closely linked to membrane properties and, more generally, to cell integrity and viability.

At higher frequencies (GHz range), corresponding to γ-dispersion, the dielectric response is mainly governed by the orientational relaxation of water molecules. In this regime, dipolar rotation of free and bound water becomes the dominant mechanism. The real part of the permittivity (ε′) decreases further, while the imaginary part (ε″) exhibits a pronounced peak associated with strong dielectric losses due to water relaxation processes.

In plant tissues, a distinct δ-dispersion is only rarely resolved experimentally. When reported in biological materials, this intermediate water-related relaxation is generally located between the β- and γ-dispersion ranges, broadly from the high-MHz region to the low-GHz region, although the exact frequency depends strongly on tissue hydration, temperature, ionic composition, and the type of macromolecular or interfacial environment [16,25,39]. Its possible molecular origins include rotational relaxation of bound or weakly bound water at cell-wall polysaccharides, proteins, membranes, starch granules, and other hydrophilic surfaces; water confined within cell-wall nanopores or intracellular compartments; hydration shells of ions and charged macromolecules; and exchange processes between bound and free water populations.

The difficulty in assigning a unique δ-dispersion arises because bound water does not have a single relaxation time. Instead, plant tissues contain a distribution of water environments, from nearly bulk-like vacuolar water to strongly restricted hydration water at charged or polymeric surfaces. As a result, the corresponding dielectric loss is broad and often appears as a shoulder rather than as a well-defined ε″ peak. Moreover, the upper tail of β-dispersion, which is associated mainly with membrane and interfacial Maxwell–Wagner polarization, can extend into the MHz region, while the low-frequency side of the γ-dispersion of free water can extend down toward the GHz region. The bound-water contribution is therefore frequently masked by the overlap between these two stronger processes. Ionic conductivity and electrode or contact effects at lower frequencies can further obscure this spectral region. For this reason, the interpretation of bound-water relaxation in plants remains challenging and usually requires broadband measurements, careful control of hydration and temperature, and multi-relaxation models such as multi-Debye or Cole–Cole representations (see Section 5).

Finally, in the high-frequency range (THz to optical frequencies), only the fastest polarization mechanisms remain active, namely electronic and atomic (ionic) polarization. All slower contributions, including α, β, and γ processes, are no longer able to follow the field variations. As a result, the dielectric constant decreases further, typically reaching values in the range of approximately 2–5.

In summary, the dielectric spectrum of plant tissues can be interpreted as a hierarchical signature of their structure: from macroscopic organization (α-dispersion) to cellular membranes (β-dispersion), molecular water dynamics (γ-dispersion), and finally intrinsic atomic and electronic polarization processes. This hierarchical framework provides the basis for interpreting the dielectric behavior observed in different plant organs and physiological conditions.

### 4.3. Dielectric Permittivity in Different Plant Organs

#### 4.3.1. Leaves

Dielectric permittivity measurements reported for leaves and plant tissues show a strong dependence on species as well as on water content and physiological and stress conditions. Studies on tea leaves under low-temperature stress [40] report a marked increase in apparent permittivity at low frequencies, associated with modifications in membrane properties and charge accumulation phenomena typical of α- and β-dispersion processes. Similarly, in olive trees, variations in electrical properties related to nitrogen status and frost tolerance [41] indicate changes in membrane stability, with a reduced dielectric response under stronger physiological stress.

For halophyte species such as *Cakile maritima* [27], impedance measurements reveal a pronounced sensitivity of permittivity to both water content and salinity, with clear modulation of β- and γ-dispersions associated with membrane polarization and intracellular water dynamics. In drought-stressed *Pinus bungeana* seedlings [42], a progressive decrease in low- and mid-frequency permittivity is observed, consistent with reduced interfacial polarization and structural alterations at the cellular level. Similarly, the complex permittivity has been investigated in *Coffea arabica* under drought stress as a function of leaf water content, allowing for the use of permittivity as a non-invasive proxy of plant water status [43].

Evidence for the importance of leaf arrangement on the plant stem has also been reported, with significant differences in the relative permittivity of winter wheat and sunflower leaves depending on their location along the stem and distance from the soil [31].

These dielectric changes are consistent with the broader physiological view that drought and water limitation affect plant performance not only by reducing tissue water content, but also by inducing stomatal closure, decreasing photosynthesis, altering root growth, modifying osmotic solute concentrations, and changing membrane transport and cellular integrity [19,21]. Therefore, dielectric permittivity in leaves can be interpreted as a non-invasive proxy for both hydration state and drought-induced physiological reorganization. In practical phenotyping, the most informative interpretation will likely combine dielectric parameters with classical plant–water indicators such as leaf water content, relative water content, water potential, stomatal conductance, and transpiration rate.

These results indicate that dielectric permittivity in leaves may provide a sensitive indicator of physiological state, reflecting membrane integrity, water status, and ionic transport processes within the tissue.

#### 4.3.2. Fruits

Dielectric permittivity in fruits and agricultural products follows trends similar to those observed in plant tissues, with a dominant dependence on water content, composition, and structural organization. Grain systems such as wheat, rice, corn, barley, oats, and grain sorghum have been extensively studied, showing a strong correlation between permittivity and moisture content and bulk density, particularly in the microwave range [26,44,45,46]. In these materials, dielectric response is largely governed by bound and free water, making permittivity a reliable indicator for non-destructive moisture and quality assessment.

For horticultural products, similar behaviors are observed despite greater structural heterogeneity. Species such as apple (*Malus domestica*, including ‘Red Delicious’), avocado (*Persea americana*), banana (*Musa *×* paradisiaca* ‘Cavendish’), cantaloupe (*Cucumis melo*), carrot (*Daucus carota*), cucumber (*Cucumis sativus*), grape (*Vitis amurensis* ‘Thompson Seedless’), orange (*Citrus* spp.), potato (*Solanum tuberosum*), watermelon (*Citrullus lanatus*), and honeydew melon have been widely investigated [44,47,48]. Across these studies, permittivity typically decreases with increasing frequency and increases with moisture content, reflecting the dominant role of liquid water in dielectric behavior.

In both grains and fruits, dielectric response is therefore primarily controlled by water-related polarization mechanisms, while structural and interfacial contributions play a secondary role compared to more complex tissues such as leaves. This makes permittivity particularly suitable for non-invasive monitoring of moisture content, ripeness, and overall product quality. Nevertheless, dielectric properties also reflect the combined influence of composition (e.g., sugars, ions, and fibers), temperature, and physiological changes occurring during ripening [49,50].

#### 4.3.3. Roots

Dielectric permittivity and related electrical properties of roots have been used as non-destructive indicators of root system architecture and physiological activity. Root tissues exhibit a strong low-frequency response dominated by interfacial polarization processes linked to ion transport at the soil–root interface and along heterogeneous conductive pathways within the root system. A comprehensive overview of dielectric and impedance-based approaches applied to root systems is provided by Liu [28], highlighting how capacitance- and impedance-based methods can capture variations in root activity, water uptake, and structural organization.

Electrical capacitance measurements have been shown to correlate with root length and overall root system size, particularly in trees [51]. This approach is based on modeling the root–soil system as a distributed capacitor, where charge accumulation occurs at root interfaces and along conductive pathways in the rhizosphere.

Similarly, in studies of arbuscular mycorrhizal colonization in maize [29], impedance and capacitance measurements reveal functional changes in root–fungus interactions. Variations in dielectric response are linked to modifications in membrane properties, ion exchange processes, and the effective conductive surface area of the root system induced by symbiosis.

Cell anatomy, root tissue structure and fluid composition play a key role in determining the polarization of roots [52].

The interpretation of root dielectric properties is particularly relevant in the context of plant water dynamics. Roots are not only conductive and polarizable biological structures, but also the main organs controlling water uptake, redistribution, and hydraulic connection between soil layers. Hydraulic redistribution can move water from wetter to drier soil regions through the root system, thereby modifying local soil moisture, root water potential, nutrient uptake, and drought tolerance [20]. These processes are expected to influence root and rhizosphere dielectric properties because they change water content, ion mobility, soil-root contact, and interfacial polarization at the root-soil boundary. Consequently, dielectric sensing of roots and rhizospheres may provide useful information not only on root biomass or architecture, but also on dynamic water redistribution and drought-response processes.

#### 4.3.4. Commonalities and Differences in Permittivity Characteristics

A systematic comparison reveals fundamental commonalities and differences in how plant organs interact with electromagnetic fields (see Table 4). The primary commonality across all organs is their universal, strict dependence on water status. In the microwave frequency region (above 1 GHz), the dielectric behavior of leaves, fruits, and stems alike is universally governed by the dipolar relaxation of free water molecules and the restriction of bound water within cellular matrices [53,54,55,56,57].

Significant differences, however, emerge at lower frequencies (sub-GHz and MHz ranges) due to distinct cellular architectures and biochemical compositions. Fruit and vegetable tissues consist of large, thin-walled parenchymal cells filled with ion-rich vacuoles and soluble sugars; this macro-structure yields massive low-frequency dielectric constants (ε′ > 300) heavily driven by bulk ionic conductivity [47,58,59]. Conversely, leaves and woody tissues possess dense cellular packing, prominent air-filled intercellular spaces, and thick cell walls (lignin/cellulose complexes). These structural barriers restrict long-range ion migration, leading to significantly lower overall ε′ values but triggering pronounced interfacial (Maxwell–Wagner) polarizations at the cell boundary levels, which are minor or absent in uniform fruit flesh [60,61,62].

## 5. Modeling Permittivity

The dielectric permittivity of materials can be modeled using different theoretical approaches depending on the complexity of the system. The simplest and most classical description is the Debye model, which assumes a single relaxation time and a homogeneous system. Under these assumptions, the complex permittivity is described by a single relaxation process characterized by a unique time constant τ, corresponding to the exponential relaxation of dipolar polarization. The Debye equation is [63]:

(1) $$\varepsilon^{*}(\omega )=\varepsilon_{\infty }+\frac{\varepsilon_{s}-\varepsilon_{\infty }}{1+j\omega \tau }$$

where $\varepsilon^{*}(\omega )$, $\varepsilon_{s}$ and $\varepsilon_{\infty }$ are the complex, static (low-frequency limit) and the permittivity at infinite frequency, respectively; ω=2πf is the angular frequency.

Separating the real and imaginary components yields:

(2) $$\varepsilon \prime (\omega )=\varepsilon_{\infty }+\frac{\varepsilon_{s}-\varepsilon_{\infty }}{1+\omega^{2}\tau^{2}}$$

and

(3) $$\varepsilon \prime \prime (\omega )=\frac{{(\varepsilon }_{s}-\varepsilon_{\infty })\omega \tau }{1+\omega^{2}\tau^{2}}$$

where $\varepsilon^{\prime }(\omega )$ and $\varepsilon^{\prime \prime }(\omega )$ are the real and imaginary part, respectively.

This model provides a good first-order approximation for simple polar liquids, such as water, and for relatively homogeneous systems where a single dominant polarization mechanism is present. In this case, the dielectric response is fully determined by one characteristic relaxation frequency ($f_{c}=\frac{1}{2\pi \tau }$) and the transition between low- and high-frequency regimes is well defined.

However, in real materials, especially biological tissues, these assumptions are generally not valid. In such systems, there is no single relaxation time, but rather a distribution of relaxation processes associated with structural and functional heterogeneity. Multiple polarization mechanisms coexist, including interfacial effects, membrane polarization, and water dipole relaxation, each characterized by different time scales.

A first physical extension of the Debye approach is the multi-Debye model, in which the total dielectric response is expressed as a sum of independent Debye-type contributions:

(4) $$\varepsilon^{*}(\omega )=\varepsilon_{\infty }+\sum_{k=1}^{n}\frac{\Delta \varepsilon_{k}}{1+j\omega \tau_{k}}$$

where *n* is the number of relaxation processes, $\Delta \varepsilon_{k}$ is the dielectric strength of the *k*-th process and $\tau_{k}$ is the relaxation time associated with the *k*-th polarization mechanism. In this framework, each term corresponds to a distinct physical process. For example, in biological tissues one can associate α-dispersion to interfacial polarization at tissue and organ level, β-dispersion to membrane-related polarization at cellular level and γ-dispersion to dipolar relaxation of water molecules. This approach improves the physical interpretability of the model, but it still assumes that each process can be described by a single well-defined relaxation time.

To account for the continuous distribution of relaxation times observed in complex systems, a more general phenomenological extension is provided by the Cole–Cole model [64]. In this formulation, an additional empirical parameter α (often called the broadening parameter) is introduced:

(5) $$\varepsilon^{*}(\omega )=\varepsilon_{\infty }+\varepsilon_{\infty }+\frac{\varepsilon_{s}-\varepsilon_{\infty }}{1+({j\omega \tau )}^{1-\alpha }}$$

where 0≤α≤1, α=0 corresponds to ideal Debye behavior and increasing values of α indicate broader distributions of relaxation times. This parameter describes the deviation from ideal Debye behavior by accounting for a distribution of relaxation times, which becomes essential when individual contributions cannot be clearly separated.

As a result, the Cole–Cole model is particularly suitable for biological tissues, where overlapping α, β, and γ dispersions produce a broad and non-ideal dielectric response. In practice, the dielectric spectrum is often described as a sum of Cole–Cole-type relaxations rather than pure Debye terms [25]:

(6) $$\varepsilon^{*}(\omega )=\varepsilon_{\infty }+\sum_{k=1}^{n}\frac{\Delta \varepsilon_{k}}{1+{\left(j\omega \tau_{k}\right)}^{1-\alpha_{k}}}$$

A useful representation for analyzing dielectric behavior is the so-called Cole–Cole diagram, in which the imaginary part of the permittivity (ε″) is plotted versus the real part (ε′). In the ideal Debye case, this plot yields a perfect semicircle [64]. In contrast, for the Cole–Cole model, the semicircle becomes depressed and broadened, reflecting the presence of a distribution of relaxation times rather than a single characteristic relaxation process.

Despite their widespread use, a major limitation of both Debye and Cole–Cole models is their phenomenological nature, as they treat heterogeneous plant tissues as effectively homogeneous media. In leaf tissue fitting, Cole–Cole models typically operate between 0.3 and 18 GHz, yielding dielectric constants (ε′) ranging from 5 to 60 depending on hydration, and successfully capturing non-Debye behavior through the broadening parameter α (0.1–0.3) [65,66]. In fruit and vegetable tissues (e.g., apple, potato), these models accurately fit broad relaxations and ionic conductivity from 10 MHz to 1.8 GHz, where ε′ drops from over 300 to below 100 as frequency increases [58,67]. However, because these parameters are empirical, they cannot explicitly account for the complex cell-to-organ anatomical geometries that dictate the actual dielectric response.

To overcome these limitations, future improvements in plant research must transition toward hierarchical multiscale dielectric modeling. Plant electrical properties naturally emerge from coupled polarization processes occurring simultaneously across multiple spatial scales—from intracellular compartments and lipid membranes to vascular bundles and whole organs. Integrating structural and anatomical data into a hierarchical framework will allow future models to address these multiscale coupling characteristics. This approach will bridge the gap between abstract fitting parameters and biophysical traits, providing direct indicators of tissue water potential, membrane integrity, and ripening-induced structural changes.

## 6. Conclusions

Dielectric permittivity provides a powerful framework to investigate the hierarchical organization of plant systems across multiple spatial and temporal scales. The dielectric response of plant tissues emerges from the combined contribution of interfacial polarization phenomena at organ/tissue level, membrane-associated processes at cellular level, and dipolar relaxation mechanisms associated with water molecules. The studies discussed in this review show that dielectric permittivity is highly sensitive to changes induced by drought, salinity, temperature stress, tissue damage, and physiological alterations. This sensitivity makes dielectric spectroscopy a promising non-invasive tool for plant phenotyping, stress monitoring, agricultural quality assessment, and root system characterization. At the same time, emerging bioelectromagnetic and plant-integrated technological applications suggest additional opportunities for integrating plant dielectric properties into wireless sensing and environmentally embedded communication systems.

Despite these advances, several limitations remain. Plant tissues are intrinsically heterogeneous systems characterized by overlapping polarization mechanisms, strong dependence on hydration conditions, and large interspecific variability. Therefore, interpretation of dielectric spectra often remains phenomenological, and direct links between dielectric behavior and specific structural or molecular traits are still incompletely understood. Standardization of experimental approaches, frequency ranges, environmental conditions, and modeling strategies will therefore be essential to improve comparability among studies.

Future developments will likely benefit from stronger integration between plant physiology, dielectric modeling, imaging approaches, and bioelectromagnetic engineering. Trait-based analyses linking dielectric behavior to anatomical, biochemical, and functional plant characteristics may help establish a more mechanistic interpretation of plant permittivity across scales. Such interdisciplinary approaches may contribute to the development of next-generation non-invasive monitoring systems for plant science, precision agriculture, and plant-integrated technologies.

Overall, dielectric permittivity may represent a unifying biophysical descriptor linking plant structure, water organization, and physiological function across multiple spatial and temporal scales.

## Figures and Tables

**Table 1:** Measurement approaches and experimental considerations for dielectric characterization of plant organs.

Plant Organ	Typical Geometry	Recommended Measurement Methods	Typical Electrode/Probe Configuration	Main Experimental Challenges	Practical Recommendations
Leaves	Thin, flat, heterogeneous lamina (0.1–2 mm thick)	Parallel-plate capacitor, open-ended coaxial probe, resonant methods	Flat electrodes or coaxial probe applied to leaf surface	Air gaps, variable thickness, veins, surface roughness, dehydration during measurement	Ensure good contact, avoid excessive pressure, measure hydration and temperature, sample multiple positions
Roots	Cylindrical, elongated structures (1–20 mm diameter)	Coaxial cells, impedance spectroscopy with contact electrodes	Ring electrodes, coaxial holders, needle electrodes	Irregular diameter, heterogeneous tissues, soil contamination, contact impedance	Clean surface carefully, standardize electrode spacing, measure diameter and moisture content
Fruits	Large, bulky, often homogeneous tissues	Open-ended coaxial probe, dielectric spectroscopy cells	Surface probe or inserted electrodes	Internal heterogeneity, skin effects, ripening stage variability	Record maturity stage, perform replicate measurements at different locations
Stems	Cylindrical, layered tissues	Impedance spectroscopy, coaxial methods	Ring electrodes, parallel electrodes on sections	Bark/cuticle effects, anisotropy, variable water content	Specify measurement orientation and tissue region
Seeds	Small, discrete particles	Resonant cavity, dielectric cell, bulk measurements	Sample holders with controlled packing density	Packing effects, air volume fraction, moisture variability	Control bulk density and moisture content

**Table 2:** Factors affecting measured dielectric permittivity in plant tissues.

Factor	Effect on Measured Permittivity	Most Affected Organs
Temperature	Alters water relaxation dynamics and conductivity	All organs
Hydration status	Strongly changes ε′ and dielectric losses, especially at microwave frequencies	Leaves, fruits
Surface roughness	Produces air gaps and contact errors	Leaves, roots
Cuticle thickness	Reduces probe coupling and electrode contact	Leaves, fruits
Sample heterogeneity	Increases measurement variability	Fruits, stems
Electrode polarization	Distorts low-frequency measurements	All contact-electrode methods

**Table 3:** Hierarchical origin of dielectric permittivity in plants across biological scales.

Hierarchical Level	Main Structures	Dominant Polarization Mechanisms	Associated Dispersion Region	Typical Frequency Range
Molecular	Water molecules, proteins, carbohydrates, lipids, ions	Electronic, atomic and dipolar polarization	γ-dispersion (water relaxation); optical polarization at higher frequencies	GHz–THz
Subcellular	Membranes, vacuoles, organelles	Maxwell–Wagner interfacial polarization, membrane charging	β-dispersion	kHz–MHz
Cellular	Cytoplasm, plasma membrane, cell wall	Membrane polarization, ionic conduction	β-dispersion	kHz–tens of MHz
Tissue	Mesophyll, xylem, phloem, epidermis, air spaces	Collective interfacial polarization and water-related relaxation	α- and β-dispersion	Hz–MHz
Organ	Leaves, stems, roots, fruits, seeds	Combined contributions of tissues and cellular compartments	α-, β-, and γ-dispersion	Hz–GHz
Whole Plant	Water transport network, organ systems	Integrated dielectric response	All dispersion regions	Hz–GHz/THz

**Table 4:** Comparative overview of dielectric permittivity characteristics in major plant organs and tissues.

Plant Organ	Dominant Polarization Mechanisms	Typical ε′ Ranges	Primary Influencing Factors	Main Applications	Key References
Leaves	Dipolar (free water) in GHz band; interfacial (Maxwell–Wagner) in MHz range.	from about 5 to 60 (highly dependent on hydration)	Relative water content (RWC), drought stress, stomatal dynamics.	Vegetation remote sensing, canopy water status, frost damage assessment.	[53,57]
Fruits and Vegetables	Bulk ionic conductivity at lower frequencies; dipolar relaxation in the GHz range.	from about 80 to >300 (low frequency); <100 (above 1 GHz)	Soluble solids content (Brix), ripening stage, temperature, tissue porosity.	Non-destructive ripeness tracking, postharvest sorting, internal quality control.	[58,59]
Roots and Stems	Interfacial polarization at cell wall-lumen boundaries; anisotropic ionic migration.	from about 5 to 150 (highly variable by wood density/species)	Sap flow rate, salinity, cambium activity, fiber orientation (anisotropy).	Standing biomass estimation, sap flow monitoring, root system mapping.	[60,61]

## Data Availability

No new data were created or analyzed in this study. Data sharing is not applicable to this article.
